# Supplementary material for: Structural basis for inhibition of the AAA-ATPase Drg1 by diazaborine
Source: Nat Commun. 2021 Jun 9;12:3483. doi: 10.1038/s41467-021-23854-x (PMC8190095; doi:10.1038/s41467-021-23854-x)
Supplement: Supplementary file 1 — Supplementary Information [file 41467_2021_23854_MOESM1_ESM.pdf]

Supplementary Information

Structural basis for inhibition of the AAA-ATPase Drg1 by diazaborine

Michael Prattes[1, 2], Irina Grishkovskaya[3], Victor-Valentin Hodirnau[4], Ingrid Rössler[1, 2], Isabella Klein[1], Christina Hetzmannseder[1], Gertrude Zisser[1], Christian Gruber[1], Karl Gruber[1, 2, 5], David Haselbach[3, *] and Helmut Bergler[1, 2, 5*]

Affiliations

[1]Institute of Molecular Biosciences, University of Graz, Graz, Austria

[2] BioTechMed-Graz, Graz, Austria

[3]Research Institute of Molecular Pathology (IMP), Vienna BioCenter, Vienna, Austria

[4]Institute of Science and Technology Austria, Klosterneuburg, Austria.

[5]Field of Excellence BioHealth - University of Graz, Graz, Austria.

*Correspondence should be addressed to D.H. or H.B.

Supplementary figures

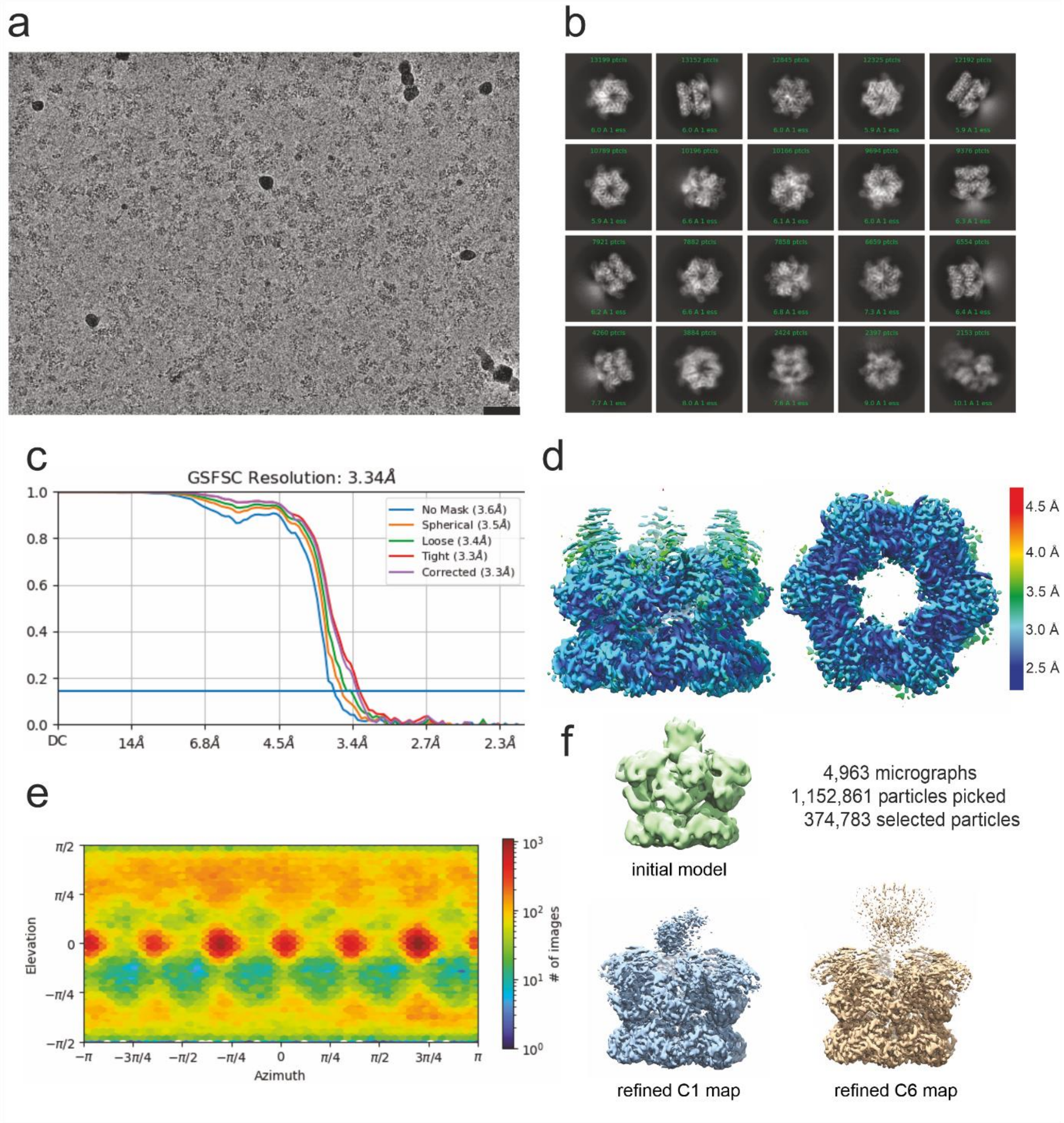

Supplementary Fig. 1: Cryo-EM data processing of the Drg1-diazaborine complex. (a) Representative raw electron micrograph from 4,963 total micrographs of the Drg1-diazaborine complex. Scalebar: 50 nm (b) Representative examples of selected 2D classes. (c) Gold-standard Fourier shell correlation (GSFSC) curves of two independently refined subsets of particles. The curve indicates a resolution of 3.34 Å at a threshold of 0.143. (d) Local resolution map. (e) Angle distribution map. (f) Particle sorting information. Based on the initial model, two maps were generated calculated either with C1 or C6 symmetry which both resulted in a highly symmetrical hexameric Drg1 maps.

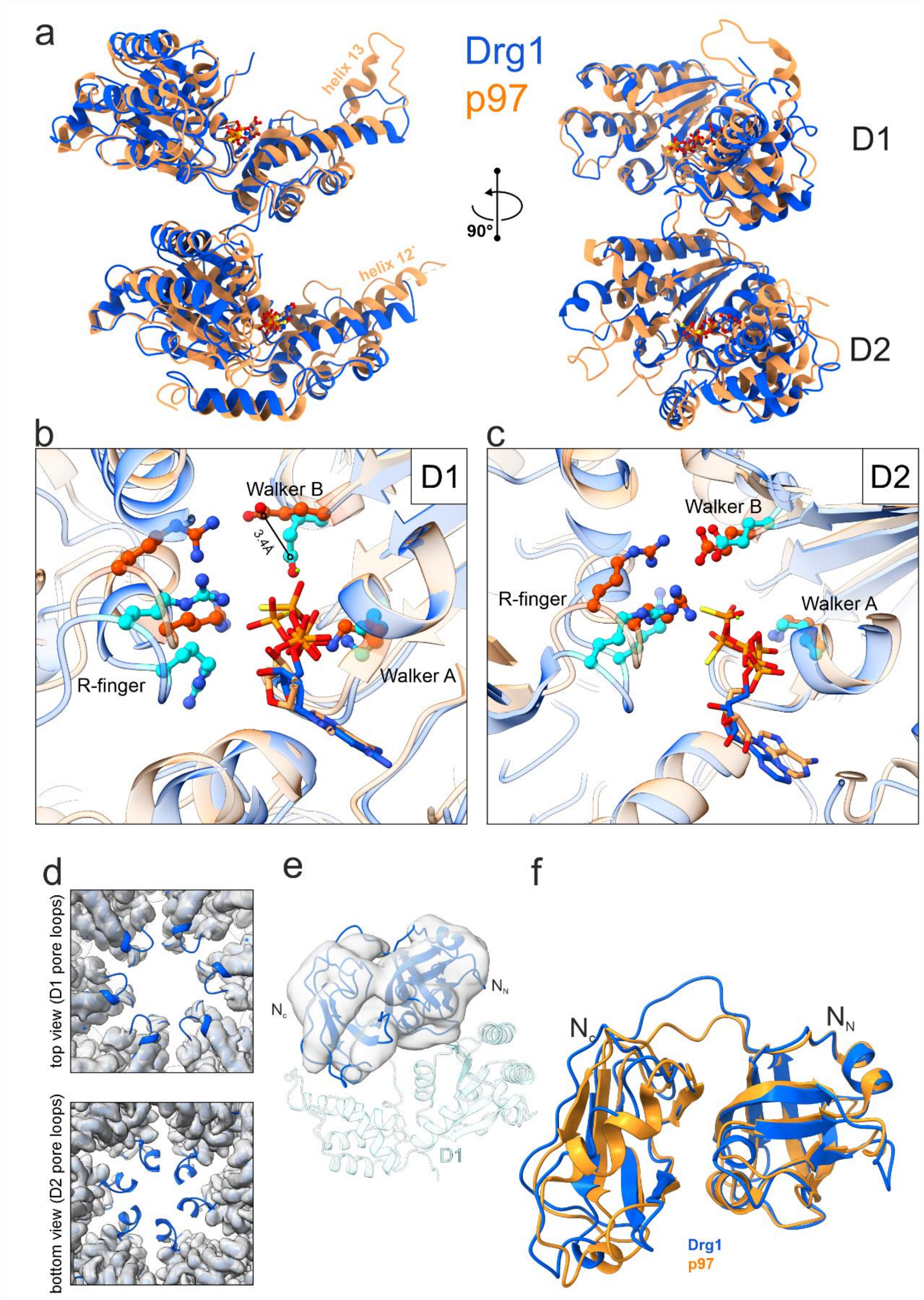

Supplementary Fig. 2: The Drg1 ATPase domains adopt the classical AAA-domain fold, highly similar to p97. (a) Superposition of the Drg1 (blue) and p97 (orange, pdb: 5FTN[1]) AAA-domains (D1 and D2) of one protomer with an overall rmsd of 3.5 Å (helix annotation according to [2]). (b and c**)** Superposition of the D1 and D2 nucleotide binding pockets of Drg1 and p97 with bound ATPγS. Essential residues of the AAA binding pocket (Walker A/B and the arginine fingers) are highlighted. (d) The pore loops in both domains are symmetrically arranged, but better resolved in the D1 domain. (e) Rigid body fitting of a Drg1 N-domain homology model into the Cryo-EM map. (f) Alignment of the Drg1 N-domain model with the p97 N-domain (pdb: 5X4L).

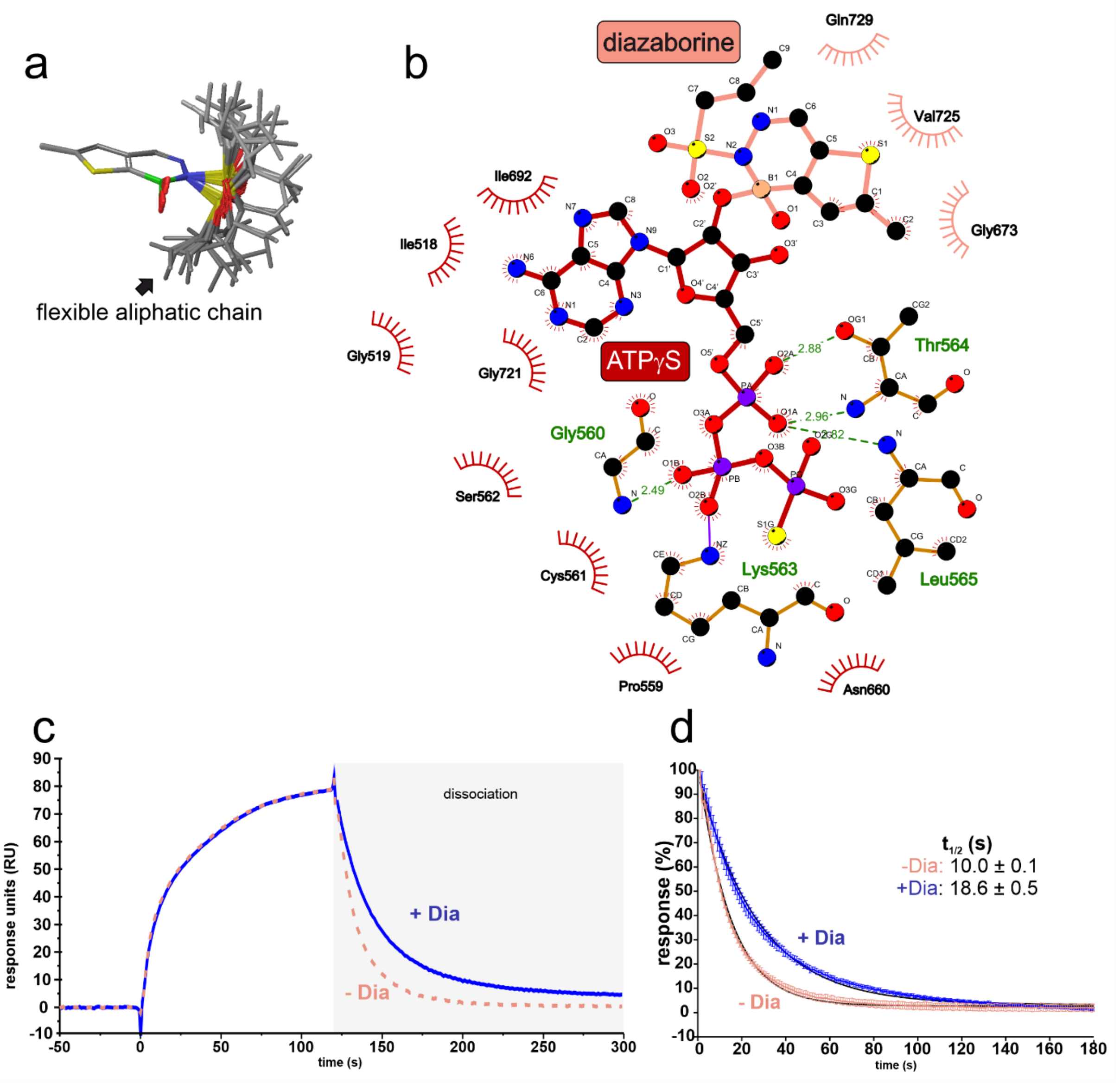

Supplementary Fig. 3: Conformational flexibility of diazaborine and its environment in the D2 nucleotide binding pocked of Drg1. (a) Conformational flexibility of used diazaborine derivative 2b18 reveals that the aliphatic chain connected to the sulphonyl group is highly flexible and can adopt various different conformations relative to the diazaborinine moiety. The conformational analysis was performed using the conformational search module of the program MacroModel from the Schrödinger molecular modeling package (www.schrodinger.com). 62 unique conformers (with a maximum rmsd of 0.5 Å) were superimposed using the atoms of the diazaborinine moiety. (b) LigPlot+ prediction of interactions between the nucleotide-inhibitor and residues of the Drg1 D2 nucleotide binding pocket. (c and d) Diazaborine slows down the dissociation of the Drg1-Rlp24C complex demonstrated by SPR. The GST-Rlp24C fragment was immobilized as a ligand on a CM5 censor chip and purified Drg1 was injected as analyte. All samples contained 1 mM ATPγS and the indicated sample additionally contained 200 µg/ml diazaborine. (c) The sensorgrams of one exemplary injection for each condition are shown. **(d)** The dissociation phases of four injections from two biological replicates (n = 4, error bars represent standard deviation) were used to calculate the half-life of the complex ($t_{1/2}$, mean and standard deviation).

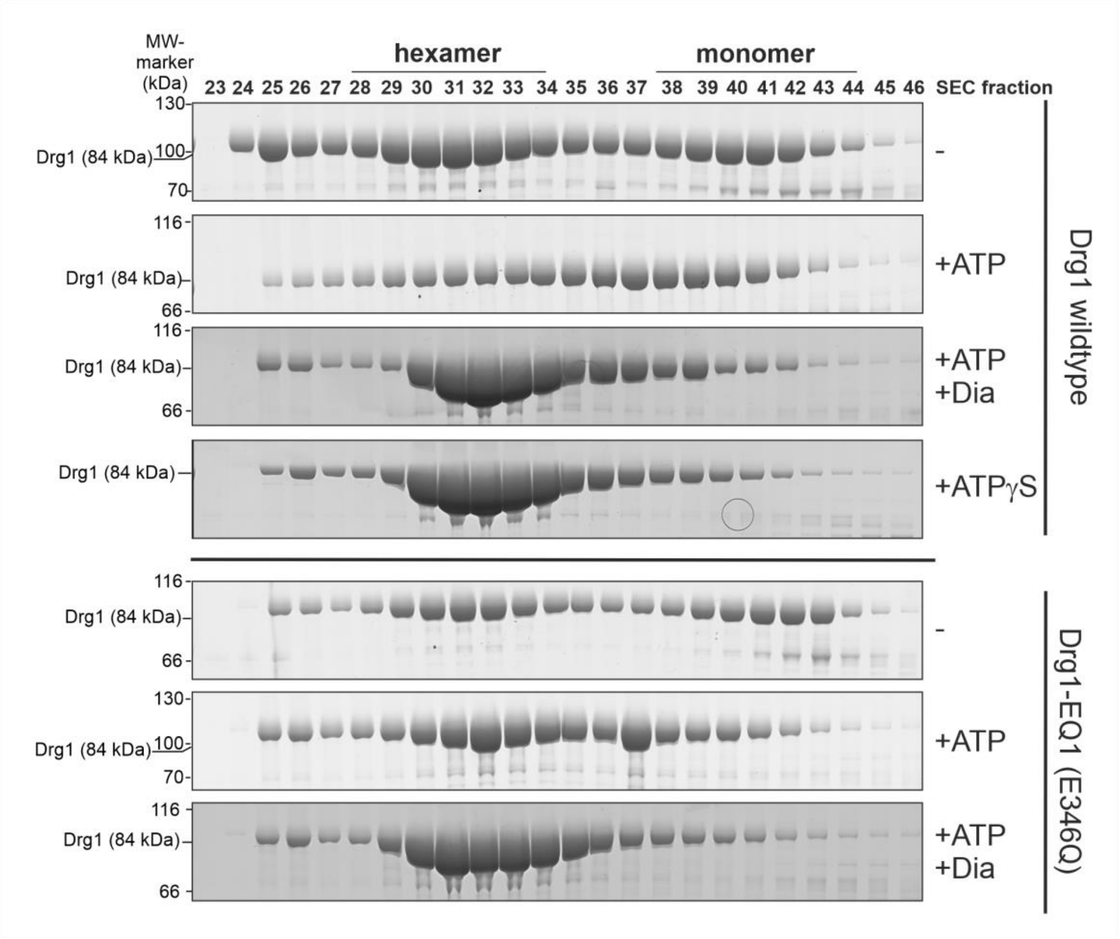

Supplementary Fig. 4: Binding of diazaborine into the D2 domain stabilizes the hexameric form of Drg1. Drg1 wildtype protein (upper panels) and the Drg1-EQ1 variant (Walker B mutation in D1, lower panels) were purified under standard conditions (-), in the presence of 5 mM ATP or non-hydrolysable ATPγS (+ATP/+ATPγS) or in the presence of 5 mM ATP and 200 µg/ml diazaborine simultaneously (+ATP +Dia). Protein samples were subjected to size exclusion chromatography to monitor the population of monomeric and hexameric protein. The shown fractions were TCA precipitated and analysed by SDS-PAGE. Gels shown are representative of n = 2 biological replicates. Uncropped gel images are provided in the Source data file. MW-marker: molecular weight marker.

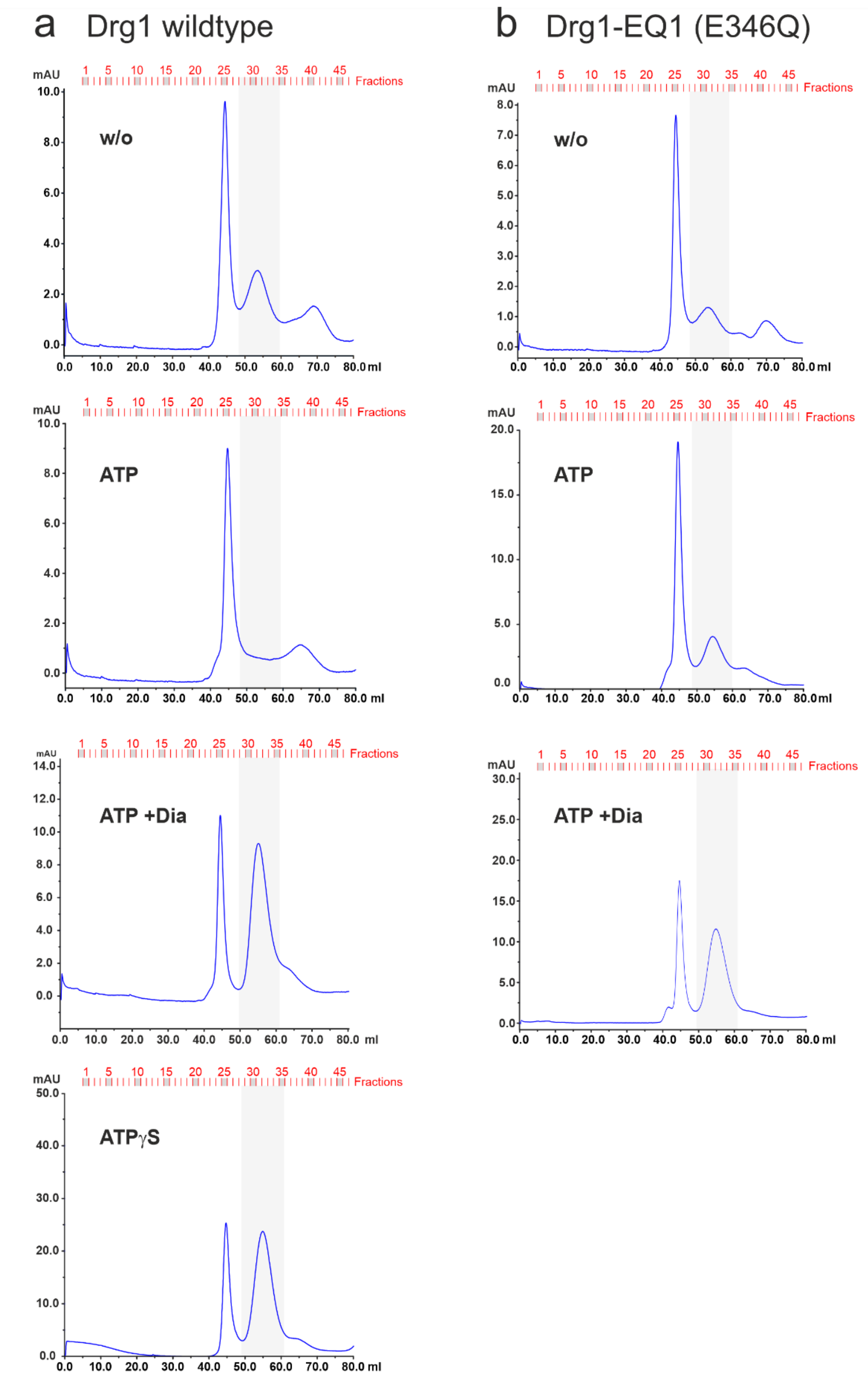

Supplementary Fig. 5: SEC profiles of the samples shown in Fig. S4. Drg1 wildtype protein (a) and the Drg1-EQ1 variant (b) were purified under standard conditions (w/o), in the presence of 5 mM ATP or slow-hydrolysable ATPγS (ATP/ATPγS) or in the presence of 5 mM ATP and 200 µg/ml diazaborine simultaneously (ATP+Dia). Protein samples were subjected to size exclusion chromatography to monitor the population of monomeric and hexameric protein. Note the different scales (mAU).

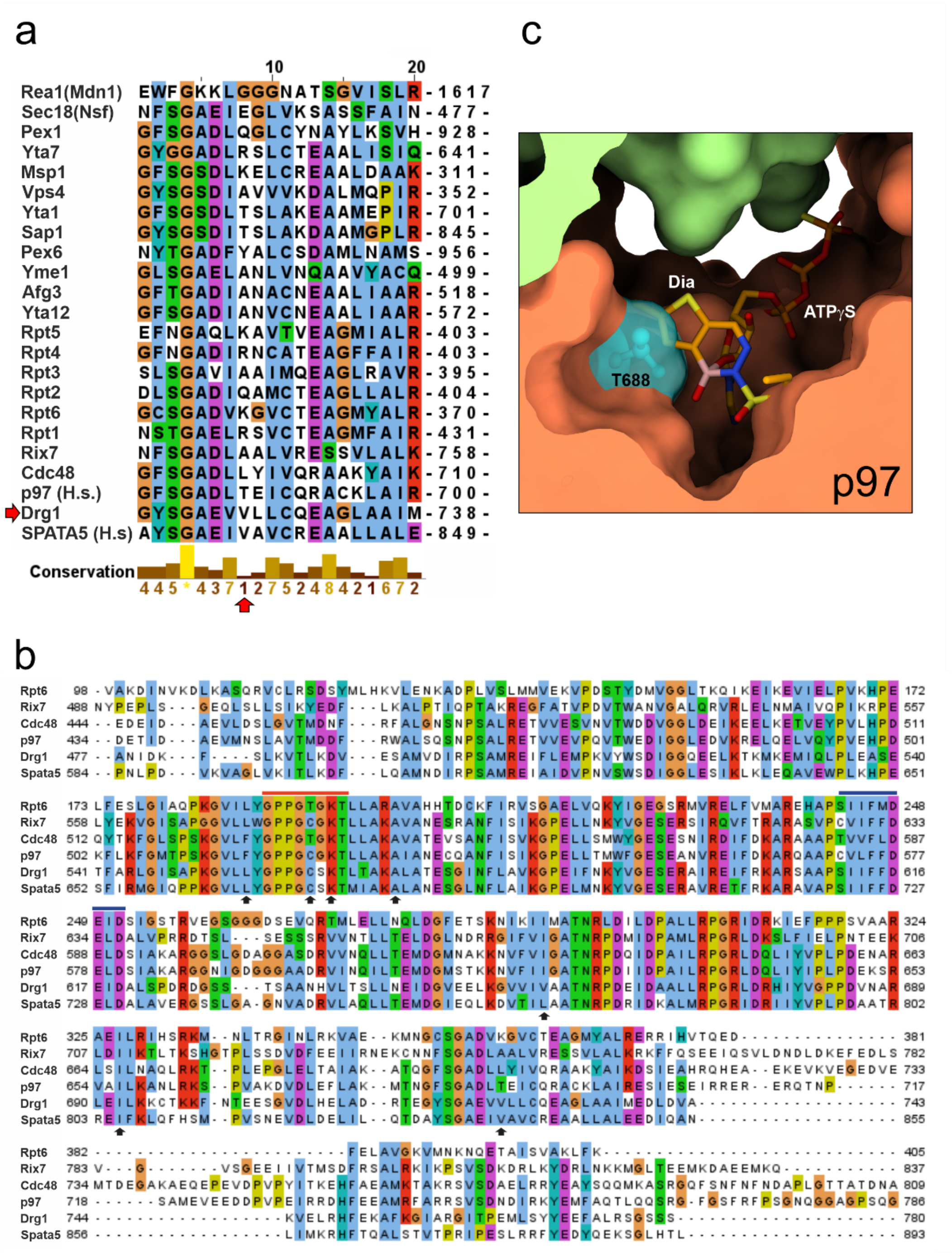

Supplementary Fig. 6: Multi-sequence alignment of Drg1 and its closest related AAA-ATPases in yeast. (a) Alignment to all AAA-ATPases in yeast showing significant homology of Drg1. The alignment focuses on the area surrounding V725 of Drg1 which is critical for diazaborine binding. A graphical representation of this alignment is shown in the main Fig 4e. The dynein-like AAA-ATPase Rea1/Mdn1from yeast and the mammalian AAA-ATPases p97 and SPATA5 were included for comparison. (b) Multi-sequence alignment of the whole Drg1 D2 domain including the related yeast AAA-ATPases Rpt6, Rix7, Cdc48 and the human proteins p97 (Cdc48 ortholog) and SPATA5 (Drg1 ortholog). Black arrows indicate the position of diazaborine resistance exchanges in Drg1. (c) Docking of the diazaborine-ATPγS adduct into the symmetric structure of p97 (pdb: 5FTJ). T688 of p97 which corresponds to V725 of Drg1 in the alignment is highlighted.

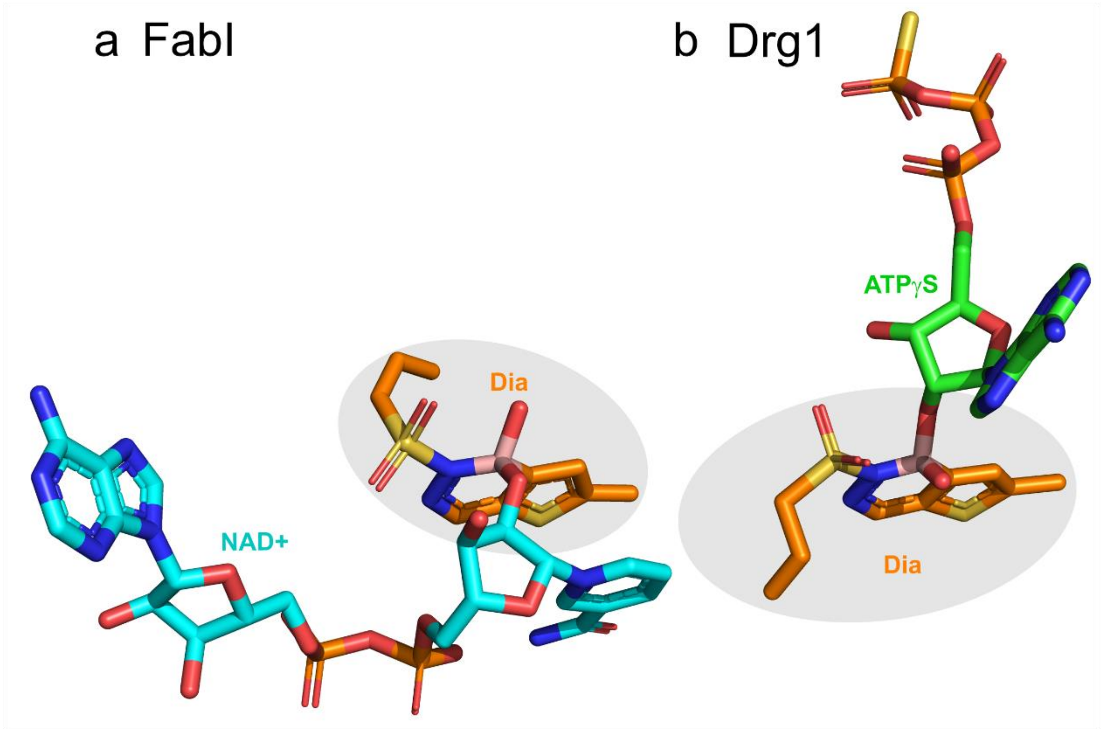

Supplementary Fig. 7: Comparison of the two nucleotide-inhibitor adducts formed in FabI (a, NAD$^+$-diazaborine, pdb: 1DFH[3]) and Drg1 (b, ATPγS-diazaborine). The adducts were superimposed using the diazaborinine moiety of the inhibitor.

Supplementary tables

Supplementary table 1: Cryo-EM data collection, refinement and validation statistics.

**Data collection and processing**	
Magnification	81000
Voltage (kV)	300
Electron exposure (e–/Å^2)	60
Defocus range (μm)	0.5-2.5
Pixel size (Å)	1.07
Symmetry imposed	C1
Initial particle images (no.)	1152861
Final particle images (no.)	237914
Map resolution (Å)<br>FSC threshold	3.4
**Model**	
**Composition (#)**	
Chains	12
Atoms	25344 (Hydrogens: 0)
Residues	Protein: 3252 Nucleotide: 0
Water	0
Ligands	TDB: 6
	AGS: 12
**Refinement**	
Bonds (RMSD)	
Length (Å) (# > 4σ)	0.007 (0)
Angles (°) (# > 4σ)	0.845 (3)
MolProbity score	2.26
Clash score	17.12
**Ramachandran plot (%)**	
Outliers	0.00
Allowed	9.04
Favored	90.96
**Rama-Z (Ramachandran plot Z-score, RMSD)**	
whole (N = 3240)	0.63 (0.15)
helix (N = 1548)	0.00 (0.13)
sheet (N = 402)	0.10 (0.28)
loop (N = 1290)	0.90 (0.18)
Rotamer outliers (%)	0.00
Cβ outliers (%)	0.00
Peptide plane (%)	
Cis proline/general	7.1/0.0
Twisted proline/general	0.0/0.0
CaBLAM outliers (%)	4.86
ADP (B-factors)	
Iso/Aniso (#)	25344/0
min/max/mean	
Protein	60.10/149.15/88.46

Nucleotide	---	
Ligand	60.88/80.76/70.77	
Water	---	
**Occupancy**		
Mean	1.00	
occ = 1 (%)	100.00	
0 < occ < 1 (%)	0.00	
occ > 1 (%)	0.00	
**Data**		
Box		
Lengths (Å)	138.86, 136.74, 98.58	
Angles (°)	90.00, 90.00, 90.00	
Supplied Resolution (Å)	3.2	
Resolution Estimates (Å)	Masked	Unmasked
d FSC (half maps; 0.143)	---	---
d 99 (full/half1/half2)	2.6/---/---	2.6/---/---
d model	2.2 2.2	
d FSC model (0/0.143/0.5)	1.8/2.0/3.6	1.8/2.0/3.6
Map min/max/mean	-0.00/2.00/0.00	
**Model vs. Data**		
CC (mask)	0.75	
CC (box)	0.71	
CC (peaks)	0.67	
CC (volume)	0.75	
Mean CC for ligands	0.81	

Supplementary table 2: Yeast and bacterial strains used in this study

Saccharomyces cerevisiae		**Source/comment**
BY4743 *drg1/DRG1*	*MATa/MATα his3/his3 leu2/leu2 met15/MET15 LYS2/lys2 ura3/ura3 YLR397c::kanMX4/YLR397c*	Euroscarf/ Heterologous expression of Drg1 variants
***DRG1* shuffle (GZAFG2)**	*MATa ura3 leu2 his3 lys2 trp1 drg1::kanMX4* [pRS316-DRG1]	[4]/spot assays
Escherichia coli		
BL21 Codon Plus (DE3)-RIPL	B F^- *ompT hsdS* (rB^- mB^-) dcm^- Tet^r *gal* (DE3) *endA* Hte [*argU ileY leuWproL* Cam^r]	Stratagene

Supplementary table 3: Plasmids

pRS316-DRG1	*CEN, 2µ, URA3*, full-length *DRG1* (+promotor and terminator)	[5]
pRS315-DRG1	*CEN, 2µ, LEU2*, full-length *DRG1* (+promotor and terminator)	[5]
pRS315-drg1-V725E (drg1-1)	*CEN, 2µ, LEU2*, full-length *drg1-V725E* (+promotor and terminator)	[4]
pRS315- drg1-V725A	*CEN, 2µ, LEU2*, full-length *drg1-V725A* (+promotor and terminator)	This study
pRS315- drg1-I692T	*CEN, 2µ, LEU2*, full-length *drg1-I692T* (+promotor and terminator)	This study
pRS315- drg1-L555F	*CEN, 2µ, LEU2*, full-length *drg1-L555F* (+promotor and terminator)	This study
pRS315- drg1-V656A	*CEN, 2µ, LEU2*, full-length *drg1-V656A* (+promotor and terminator)	This study
pCUP1-DRG1 (pAZ7)	URA3, AmpR, ColE1, CUP1, GST-tag, Prescission protease site, full-length *DRG1* (wildtype)	[6]
pCUP1-drg1-V725E (drg1-1)	URA3, AmpR, ColE1, CUP1, GST-tag, Prescission protease site, full-length *drg1-1* (wildtype)	[6]
pCUP1-drg1-V725A	URA3, AmpR, ColE1, CUP1, GST-tag, Prescission protease site, full-length *drg1-V725A*	This study
pCUP1-drg1-I692T	URA3, AmpR, ColE1, CUP1, GST-tag, Prescission protease site, full-length *drg1-I692T*	This study
pCUP1-drg1-L555F	URA3, AmpR, ColE1, CUP1, GST-tag, Prescission protease site, full-length *drg1-L555F*	This study
pCUP1-drg1-V656A	URA3, AmpR, ColE1, CUP1, GST-tag, Prescission protease site, full-length *drg1-V656A*	This study
pET32a-RLP24C	AmpR, N-terminal tags (TrX-tag, HIS6-tag, S-tag), *RLP24C* (base pairs 441–599, amino acids 147-199)	[5]

Supplementary table 4: software and algorithms

Software/Algorithm	citation	source
Cavman		Innophore GmbH, www.innophore.com
Coot v0.9.2	7	https://www2.mrc-lmb.cam.ac.uk/personal/pemsley/coot/
Corbett Rotor-Gene Series 6000 software v1.7		https://www.gene-quantification.de/corbett/
Cryosparc v3.0	8–10	https://cryosparc.com/
DeepEMhancer	11	https://github.com/rsanchezgarc/deepEMhancer
GraphPad Prism v3.03		https://www.graphpad.com/scientific-software/prism/
Ligplot+ v2.2	12	https://www.ebi.ac.uk/thornton-srv/software/LigPlus/
Ligsite algorithm	13	
MacroModel		Schrödinger Release 2021-1: Schrödinger, LLC https://www.schrodinger.com/products/macromodel
Microsoft Excel 2019		https://www.microsoft.com/de-at/microsoft-365/excel
PHENIX suite v1.18.2-3874	14	https://www.phenix-online.org
Phyre2	15	http://www.sbg.bio.ic.ac.uk/~phyre2/html/page.cgi?id=index
RELION v3.0	16	http://www2.mrc-lmb.cam.ac.uk/relion/index.php/Main_Page
Rosetta v3.0		https://www.rosettacommons.org/
SerialEM v3.8	17	https://bio3d.colorado.edu/SerialEM/
UCSF Chimera v.1.15	18	http://www.cgl.ucsf.edu/chimera/
UCSF ChimeraX v1.1.1	19	https://www.rbvi.ucsf.edu/chimerax/
UCSF pyem v0.5	20	https://zenodo.org/record/3576630
Unicorn™ 6.4 (SEC)		GE Healthcare Life Sciences/Cytiva https://www.cytivalifesciences.com/en/us/shop/chromatography/software/unicorn-6-control-software-p-00772

Supplementary table 5: primers used in this study

Name	Sequence 5´-3´	use
Drg1_SacI_fwd	TATAGAGCTCATTTATTCTCAATGTGAGTCATGT	Random mutagenesis of *DRG1*
Drg1_SpeI_rev	ATATAACTAGTTCCAATTTGCTA	
Drg1_Sal_fwd	TTCTGTCGACCCTGCTCTCAGGAGA	Subcloning of mutant *drg1* alleles into the pCUP1 expression vector
Drg1_Not_rev	AAAAGCGGCCGCTTACGAAGATGAACCGCTTCTTAG	
DRGseq501	AAAAACAAAAGCAGGTGA	Sequencing
DRGseqR835	GAGGGGGAGAAACACCAA	
DRGseq1501	AGCGCAATGAGAGAAATC	

Supplementary references

1. Banerjee, S. *et al.* 2.3 Å resolution cryo-EM structure of human p97 and mechanism of allosteric inhibition. *Science* **351**, 871–875 (2016).
2. DeLaBarre, B. & Brunger, A. T. Complete structure of p97/valosin-containing protein reveals communication between nucleotide domains. *Nature Structural Biology* **10**, 856–863 (2003).
3. Baldock, C. *et al.* A mechanism of drug action revealed by structural studies of enoyl reductase. *Science* **274**, 2107–2110 (1996).
4. Loibl, M. *et al.* The drug diazaborine blocks ribosome biogenesis by inhibiting the AAA-ATPase Drg1. *J. Biol. Chem.* **289**, 3913–3922 (2014).
5. Kappel, L. *et al.* Rlp24 activates the AAA-ATPase Drg1 to initiate cytoplasmic pre-60S maturation. *J. Cell Biol.* **199**, 771–782 (2012).
6. Zakalskiy, A. *et al.* Structural and enzymatic properties of the AAA protein Drg1p from Saccharomyces cerevisiae. Decoupling of intracellular function from ATPase activity and hexamerization. *J. Biol. Chem.* **277**, 26788–26795 (2002).
7. Emsley, P. & Cowtan, K. Coot: model-building tools for molecular graphics. *Acta Crystallogr D Biol Crystallogr* **60**, 2126–2132 (2004).
8. Punjani, A., Rubinstein, J. L., Fleet, D. J. & Brubaker, M. A. cryoSPARC: algorithms for rapid unsupervised cryo-EM structure determination. *Nature Methods* **14**, 290–296 (2017).
9. Punjani, A., Zhang, H. & Fleet, D. J. Non-uniform refinement: adaptive regularization improves single-particle cryo-EM reconstruction. *Nature Methods* **17**, 1214–1221 (2020).
10. Punjani, A. & Fleet, D. J. 3D Variability Analysis: Resolving continuous flexibility and discrete heterogeneity from single particle cryo-EM. *bioRxiv* 2020.04.08.032466 (2021) doi:10.1101/2020.04.08.032466.
11. Sanchez-Garcia, R. *et al.* DeepEMhancer: a deep learning solution for cryo-EM volume post-processing. *bioRxiv* 2020.06.12.148296 (2020) doi:10.1101/2020.06.12.148296.
12. Laskowski, R. A. & Swindells, M. B. LigPlot+: multiple ligand-protein interaction diagrams for drug discovery. *J Chem Inf Model* **51**, 2778–2786 (2011).

13. Hendlich, M., Rippmann, F. & Barnickel, G. LIGSITE: automatic and efficient detection of potential small molecule-binding sites in proteins. *J Mol Graph Model* **15**, 359–363, 389 (1997).

14. Adams, P. D. *et al.* PHENIX: a comprehensive Python-based system for macromolecular structure solution. *Acta Crystallogr D Biol Crystallogr* **66**, 213–221 (2010).

15. Kelley, L. A., Mezulis, S., Yates, C. M., Wass, M. N. & Sternberg, M. J. E. The Phyre2 web portal for protein modeling, prediction and analysis. *Nat. Protocols* **10**, 845–858 (2015).

16. Scheres, S. H. W. RELION: implementation of a Bayesian approach to cryo-EM structure determination. *J Struct Biol* **180**, 519–530 (2012).

17. Mastronarde, D. N. Automated electron microscope tomography using robust prediction of specimen movements. *J Struct Biol* **152**, 36–51 (2005).

18. Pettersen, E. F. *et al.* UCSF Chimera--a visualization system for exploratory research and analysis. *J Comput Chem* **25**, 1605–1612 (2004).

19. Goddard, T. D., Huang, C. C. & Ferrin, T. E. Visualizing density maps with UCSF Chimera. *Journal of Structural Biology* **157**, 281–287 (2007).

20. Daniel Asarnow, Eugene Palovcak & Yifan Cheng. *asarnow/pyem: UCSF pyem v0.5*. (Zenodo, 2019). doi:10.5281/zenodo.3576630.
