# Supplementary material for: Structural basis for inhibition of the AAA-ATPase Drg1 by diazaborine
Source: Nat Commun. 2021 Jun 9;12:3483. doi: 10.1038/s41467-021-23854-x (PMC8190095; doi:10.1038/s41467-021-23854-x)
Supplement: Supplementary file 3 — Description of Additional Supplementary Files [file 41467_2021_23854_MOESM3_ESM.pdf]

Description of Additional Supplementary Files

File Name: Supplementary Movie 1
Description: **Conformational flexibility of the Drg1- diazaborine complex.** While the N- and D1 domain show coordinated movements, the D2 domain remained in a rigid state. The video shows a volume series generated by 3D variability analysis calculated in Cryosparc with C1 symmetry and filtered to 7 Å resolution. The Volume series was animated in Chimera and the fuzzy density above the pore entrance (Fig. S1) was removed for clarity.
