# Supplementary material for: Structural basis for inhibition of the AAA-ATPase Drg1 by diazaborine
Source: Nat Commun. 2021 Jun 9;12:3483. doi: 10.1038/s41467-021-23854-x (PMC8190095; doi:10.1038/s41467-021-23854-x)
Supplement: Supplementary file 5 — Reporting Summary [file 41467_2021_23854_MOESM5_ESM.pdf]

nature research

Corresponding author(s): Helmut Bergler and David Haselbach

Last updated by author(s): May 19, 2021

Reporting Summary

Nature Research wishes to improve the reproducibility of the work that we publish. This form provides structure for consistency and transparency in reporting. For further information on Nature Research policies, see our Editorial Policies and the Editorial Policy Checklist.

Statistics

For all statistical analyses, confirm that the following items are present in the figure legend, table legend, main text, or Methods section.

n/a	Confirmed	
☐	☒	The exact sample size (*n*) for each experimental group/condition, given as a discrete number and unit of measurement
☐	☒	A statement on whether measurements were taken from distinct samples or whether the same sample was measured repeatedly
☒	☐	The statistical test(s) used AND whether they are one- or two-sided<br>*Only common tests should be described solely by name; describe more complex techniques in the Methods section.*
☒	☐	A description of all covariates tested
☒	☐	A description of any assumptions or corrections, such as tests of normality and adjustment for multiple comparisons
☐	☒	A full description of the statistical parameters including central tendency (e.g. means) or other basic estimates (e.g. regression coefficient) AND variation (e.g. standard deviation) or associated estimates of uncertainty (e.g. confidence intervals)
☒	☐	For null hypothesis testing, the test statistic (e.g. *F*, *t*, *r*) with confidence intervals, effect sizes, degrees of freedom and *P* value noted<br>*Give P values as exact values whenever suitable.*
☒	☐	For Bayesian analysis, information on the choice of priors and Markov chain Monte Carlo settings
☒	☐	For hierarchical and complex designs, identification of the appropriate level for tests and full reporting of outcomes
☒	☐	Estimates of effect sizes (e.g. Cohen's *d*, Pearson's *r*), indicating how they were calculated

Our web collection on statistics for biologists contains articles on many of the points above.

Software and code

Policy information about availability of computer code

Data collection	DSF data were collected on a Corbett Rotor-Gene series 6000 thermocycler with the associated software (Rotor-Gene series 6000 software v1.7). Raw data (OD600) of the ATPase activity measurements were collected on a Tecan plate reader and an associated Microsoft excel macro plugin (XFluor4 v4.51). The SEC data were collected using the Unicorn™ software v6.4 (GE Healthcare/Cytiva). Cryo-EM data were recorded with SerialEM v3.8.
Data analysis	DSF data were analyzed using the Rotorgene series 6000 software v1.7 to calculate the dF/dT curves. For the calculation of the MIC for diazaborine as well as the analysis of biochemical data (including ATPase activity measurements, inhibitor binding affinities), Microsoft Excel 2019 and the Graphpad Prism software v 3.03 were used.<br>Software for structure analysis: Cavman (Innophore GmbH, www.innophore.com), Coot v0.9.2, Cryosparc v3.0, DeepEMhancer (Sanchez-Garcia et al. 2020, https://github.com/rsanchezgarc/deepEMhancer), Ligplot+ v2.2, LigSite algorithm (Hendlich et al., 1997), MacroModel (Schrödinger Release 2021-1), PHENIX suite v1.18.2-3874, Phyre2 (v2.0), RELION v3.0, Rosetta v3.0, UCSF Chimera v.1.15, UCSF ChimeraX v1.1.1, UCSF pyem v0.5.

For manuscripts utilizing custom algorithms or software that are central to the research but not yet described in published literature, software must be made available to editors and reviewers. We strongly encourage code deposition in a community repository (e.g. GitHub). See the Nature Research guidelines for submitting code & software for further information.

Data

Policy information about availability of data

All manuscripts must include a data availability statement. This statement should provide the following information, where applicable:

- Accession codes, unique identifiers, or web links for publicly available datasets
- A list of figures that have associated raw data
- A description of any restrictions on data availability

Structural data generated in this study were deposited in the PDB (accession code: 7NKU, https://www.rcsb.org/structure/7NKU) and EMDB (accession code: 12448, https://www.ebi.ac.uk/pdbe/entry/emdb/EMD-12448) databases. The raw data (unprocessed micrographs) are deposited in the EMPIAR database (accession code: 10717, https://www.ebi.ac.uk/pdbe/emdb/empiar/entry/10717). Additional published datasets used for analysis in this study are also available from the pdb: 6OPC (https://www.rcsb.org/structure/6OPC), 5FTJ (https://www.rcsb.org/structure/5FTJ), 5FTN (https://www.rcsb.org/structure/5FTN) and 5X4L (https://www.rcsb.org/structure/5X4L). The Cdc48-based homology model of Drg1 is available upon request from the authors. Source data for the graphs and calculated parameters in figures 2d, 2e, 4d, 5a, 5b and S3d as well as the uncropped gel scans corresponding to Fig. S4 are provided with this paper as Source data file (source_data.xlsx).

Field-specific reporting

Please select the one below that is the best fit for your research. If you are not sure, read the appropriate sections before making your selection.

- [x] Life sciences
- [ ] Behavioural & social sciences
- [ ] Ecological, evolutionary & environmental sciences

For a reference copy of the document with all sections, see nature.com/documents/nr-reporting-summary-flat.pdf

Life sciences study design

All studies must disclose on these points even when the disclosure is negative.

Sample size	Sample sizes (n) are supplied in the figure legends in the main text or the supplementary information file. No mathematical sample size calculation was performed. All biochemical and yeast growth experiments were performed with multiple biological and technical replicates to allow estimation of the distribution of the data. Sample sizes are based on preliminary and published studies (Kappel et al., 2012, Loibl et al., 2014, Prattes et al., 2017) and were determined by number of replicates necessary to ensure reproducibility. Detailed information for the individual experiments including sample size and replicates are stated in the figure legends, the methods section as well as the source data file provided along with this paper.
Data exclusions	No datasets for the biochemical measurements and yeast growth experiments were excluded.
Replication	For all biochemical measurements, 2-4 biological replicates were tested, each measured with at least two technical replications. Detailed information for the individual experiments are stated in the figure legends, the methods section as well as the source data file provided along with this paper.
Randomization	Since only structural biology, biochemical and yeast growth experiments were performed, randomization was not applied
Blinding	Blinding was not applicable for the in vitro and in vivo experiments in this study since no human or animal subjects were involved. For the biochemical assays and yeast growth experiments in this study, blinding was not applicable, since the same investigator was doing group allocation during data collection and/or analysis.

Reporting for specific materials, systems and methods

We require information from authors about some types of materials, experimental systems and methods used in many studies. Here, indicate whether each material, system or method listed is relevant to your study. If you are not sure if a list item applies to your research, read the appropriate section before selecting a response.

Materials & experimental systems

n/a	Involved in the study	
x		Antibodies
x		Eukaryotic cell lines
x		Palaeontology and archaeology
x		Animals and other organisms
x		Human research participants
x		Clinical data
x		Dual use research of concern

Methods

n/a	Involved in the study	
x		ChIP-seq
x		Flow cytometry
x		MRI-based neuroimaging
